# A Single Amino Acid Substitution in the Transmembrane Domain of Glycoprotein H Functionally Compensates for the Absence of gL in Pseudorabies Virus

**DOI:** 10.3390/v16010026

**Published:** 2023-12-22

**Authors:** Melina Vallbracht, Marina Schnell, Annemarie Seyfarth, Walter Fuchs, Richard Küchler, Thomas C. Mettenleiter, Barbara G. Klupp

**Affiliations:** 1 Institute of Molecular Virology and Cell Biology, Friedrich-Loeffler-Institut, Federal Research Institute for Animal Health, 17493 Greifswald-Insel Riems, Germany; melina.vallbracht@bioquant.uni-heidelberg.de (M.V.); richard.kuechler@fli.de (R.K.);; 2 Schaller Research Groups, Department of Infectious Diseases, Virology, Heidelberg University Hospital, 69120 Heidelberg, Germany; 3 Department of Hematology, Oncology and Tumor Immunology, CBF, Charité—Universitätsmedizin, Corporate Member of Freie Universität Berlin und Humboldt—Universität zu Berlin, 12200 Berlin, Germany

**Keywords:** herpesvirus, pseudorabies virus, PrV, virus entry, membrane fusion, glycoprotein, gB, gH/gL, in vitro fusion assay, transmembrane domain

## Abstract

Herpesvirus entry requires the coordinated action of at least four viral glycoproteins. Virus-specific binding to a cellular receptor triggers a membrane fusion cascade involving the conserved gH/gL complex and gB. Although gB is the genuine herpesvirus fusogen, it requires gH/gL for fusion, but how activation occurs is still unclear. To study the underlying mechanism, we used a gL-deleted pseudorabies virus (PrV) mutant characterized by its limited capability to directly infect neighboring cells that was exploited for several independent serial passages in cell culture. Unlike previous revertants that acquired mutations in the gL-binding N-terminus of gH, we obtained a variant, PrV-ΔgLPassV99, that unexpectedly contained two amino acid substitutions in the gH transmembrane domain (TMD). One of these mutations, I662S, was sufficient to compensate for gL function in virus entry and in in vitro cell–cell fusion assays in presence of wild type gB, but barely for cell-to-cell spread. Additional expression of receptor-binding PrV gD, which is dispensable for cell–cell fusion mediated by native gB, gH and gL, resulted in hyperfusion in combination with gH V99. Overall, our results uncover a yet-underestimated role of the gH TMD in fusion regulation, further shedding light on the complexity of herpesvirus fusion involving all structural domains of the conserved entry glycoproteins.

## 1. Introduction

Herpesvirus entry requires the concerted action of at least four different glycoproteins. Whereas receptor binding is mediated by virus-specific glycoproteins (g), e.g., gD in the alphaherpesviruses herpes simplex viruses (HSV-1/-2, *Simplexvirus humanalpha1/2*) and pseudorabies virus (PrV, *Varicellovirus suidalpha1*), actual fusion of the membranes is controlled by a conserved machinery composed of the heterodimeric gH/gL complex and gB. While the role of gB as a genuine herpesviral fusogen is well established, fusion activation by the heterodimeric gH/gL complex is still enigmatic (reviewed in [1,2,3]).

The HSV-1 gH/gL complex was shown to interact with cellular integrin receptors, and binding is thought to trigger the dissociation of gL from the complex, thereby promoting gH activation [4]. These data suggest that gL may act as a negative regulator that maintains gH in an inactive state until it is released upon receptor binding. Despite the high structural similarity to HSV-1, PrV gH does not comprise an integrin-binding motif [5], and gL-dissociation has not yet been observed, indicating that gH/gL-activation through integrin-binding might be an accessory or non-conserved feature. Nevertheless, other yet-unidentified gH/gL-specific cellular receptors might exist.

gL is a small glycoprotein (approximately 20kDa in PrV) that lacks a transmembrane domain (TMD), but is associated with the membrane via interaction with the N-terminal domain of gH. In many herpesviruses, processing and correct localization of gH is dependent on gL, and it was originally assumed that gL acts as a chaperone to assist correct gH folding and transport [6]. Conversely, in PrV gH is incorporated into the virion envelope in the absence of gL, but mutants lacking gL are nevertheless deficient in entry, showing a role for gL beyond chaperoning [7].

In addition to structural analyses and targeted mutagenesis of the core fusion glycoproteins, we repeatedly used a more indirect approach to analyze their function. PrV gL is not absolutely essential for direct cell-to-cell transmission, and small foci of infected cells are formed in its absence. We used this limited spreading capacity of PrV-ΔgL for reversion analyses by repeated co-seeding of infected with non-infected cells [8,9]. The selected revertants offered different and often unexpected answers for compensation of the missing gL function. In the first passaging experiment, the revertant expressed a hybrid protein consisting of the receptor-binding region of gD fused to a gH lacking the gL–interaction domain [9]. This multifunctional gD-gH hybrid protein simultaneously compensated for the functions of gD, gH and gL, thereby reducing the complexity of herpesviral membrane fusion from four to only two proteins [10]. An attempt to generate a similar functional gD-gH hybrid protein for HSV-1 was not successful [11], but HSV-2 gH mutants lacking N-terminal residues were transported and processed in the absence of gL [12], thereby allowing differentiation of the transport/chaperone function and the function in gH/gL triggered fusion. Furthermore, a complex containing N-terminally truncated HSV-2 gH (gHΔ48/gL) is able to induce low level constitutive membrane fusion by gB in the absence of gD and/or a cellular receptor indicating that gHΔ48/gL constitutes a partially activated form of this glycoprotein complex [13].

A gH mutant derived from a second PrV-ΔgL passaging experiment contained only two amino acid substitutions in the N-terminal part of gH (L70P and W103R), which were found sufficient to mediate gL-independent cell–cell fusion [8]. In addition, this revertant encoded a hyperfusogenic gB, which was negatively regulated by an inhibitory mutation in gD pointing to a finely balanced, but also flexible, interaction between the components of the fusion machinery. Altogether, these data indicated that neither gL nor the gL-binding domain in gH are absolutely required for membrane fusion.

Soluble HSV-1 gH/gL in combination with gB and gD was shown to induce in vitro membrane fusion, indicating that the ectodomains of gB and gH/gL directly interact to trigger the fusion reaction [14]. In addition, cell–cell fusion in trans by gB and gH/gL expressed in different cells was observed for HSV-1 [14] and for human cytomegalovirus [15]. This implies that the gH transmembrane and intracytoplasmic domains play no essential role in fusion, and that activation takes place solely via the ectodomains. Conversely, it could be shown that the TMD and the cytoplasmic tail of gH cannot be substituted by heterologous sequences, indicating a functional role in membrane fusion [16,17,18,19,20,21]. 

For the HSV-1 gB and gH cytoplasmic domains (CTD), it has been proposed that gB is locked in a prefusion form by its highly structured CTD until the tight interaction with the membrane is released by the gH CTD, thereby allowing for allosteric unfolding of the gB ectodomain [22,23]. However, for PrV, we could show that the gH CTD is dispensable for virus entry, although in vitro fusion activity and viral titers were reduced in the presence of a truncated gH CTD [20]. Furthermore, even the fusion regulation by the CTD of PrV gB can be overcome by a single amino acid exchange in the gB ectodomain (N735S) [24], indicating that fusion is safeguarded by more than one mechanism.

While the HSV-1 gH TMD could functionally substitute for the TMD in PrV gH [20], this was not the case for the Epstein–Barr virus gH TMD in HSV-1 gH and vice versa [16], further supporting an important and apparently specific function of the TMD beyond anchoring the protein in the membrane.

Here, we report on a gL-independently acting PrV gH variant (gH V99) comprising two amino acid substitutions (A651T, I662S) in the predicted TMD. In contrast to in vitro cell–cell fusion mediated by wild type gH and gB, where gD has no major effect, the addition of gD resulted in hyperfusion in combination with gH V99. The substitution of isoleucine at position 662 to serine was sufficient to compensate for loss of gL during virus entry and in cell–cell fusion assays, but did not restore wild type-like direct cell-to-cell transmission. Efficient gL-independent in vitro cell–cell fusion was also observed with other amino acids at position 662, e.g., alanine, cysteine and threonine. However, several amino acids with bulky side chains, arginine or tyrosine at position 662, resulted in a nonfunctional gH pointing to functionally important intramembrane interactions.

## 2. Materials and Methods

### 2.1. Cells and Viruses

Rabbit kidney (RK13) and RK13-gH/gL cells [25] were grown in Eagle’s minimum essential medium (MEM) supplemented with 10% fetal bovine serum (FBS) at 37 °C and 5% CO_2_. PrV strain Kaplan (PrV-Ka) [26] and a variant expressing gfp in the non-essential gG gene locus, PrV-ΔgGgfp [27], were propagated on RK13 cells. PrV-∆gLgfp, in which gL-specific sequences were substituted by the gfp expression cassette [25], was grown on RK13-gH/gL cells.

### 2.2. Passaging Experiment

RK13 cells were infected with phenotypically complemented PrV-ΔgLgfp at a MOI of 0.1. When the cells showed CPE, they were trypsinized and co-seeded with fresh RK13 cells. The supernatants were checked for infectivity on RK13 cells and, after stably reaching titers of approximately 10^4^ plaque forming units (PFU)/ml, only the supernatants were used for further passages. From the supernatant of the 99th passage, six different plaques were picked and the isolated viruses were initially characterized. Since no gross differences were observed in these screens, one single plaque isolate designated as PrV-ΔgLPassV99 was further analyzed.

### 2.3. In Vitro Replication Properties

RK13 cells were infected with the wild type-like recombinant PrV-ΔgGgfp or the different other gfp-expressing mutants for 1 h at 4 °C. After temperature shift and an additional hour at 37 °C, non-penetrated virus was inactivated by low-pH treatment [28]. Infected cells and supernatants were harvested at different times thereafter and progeny virus titers were determined on RK13 cells. For plaque size measurement RK13 cells were infected with the different mutants and the plaque diameter of 15 plaques each was measured. The mean plaque size of PrV-ΔgGgfp was set as 100%, and relative sizes were calculated for the other mutants. Mean values and standard deviations from at least three independent assays were calculated.

### 2.4. Sequencing and Site Directed Mutagenesis

PCR amplification and sequencing were used to identify mutations in genes encoding the entry glycoproteins as described [8]. Site-directed mutagenesis (QuikChange II XL kit; Agilent, Waldbronn, Germany) was applied to introduce mutations into the cloned gH gene using primers given in Table 1 and plasmids pcDNA-gH [10] or pcDNA-gHKDE [29] as templates. Correct mutagenesis was verified by sequencing. Geneious software, version 10.2.5 (Biomatters), was used for sequence analyses.

### 2.5. Transient Transfection-Based Fusion Assay

Fusion activity was tested in transfection based in vitro fusion assays as described [30]. Briefly, approximately 1.8 × 10^5^ RK13 cells per well were seeded into 24-well plates. On the following day, cells were co-transfected with 200 ng each of plasmids encoding gD, gB, gH, and optionally gL using Lipofectamine 2000 (Thermo Fisher Scientific, Dreieich, Germany) as recommended by the manufacturer. A plasmid encoding the enhanced green fluorescent protein (peGFP-N1; Clontech, Heidelberg, Germany) was included as marker for easy visualization of transfected cells and syncytia. Transfection assays were evaluated after 18–24 h by measuring the area and number of green fluorescing syncytia within 5 or 10 fields of view using a fluorescence microscope (Nikon Eclipse Ti-S) as described [30]. Fusion activity was determined using the Nikon NIS-Elements imaging software, version 4.0. All experiments were performed at least in triplicate, and average values with the corresponding standard deviations were calculated.

### 2.6. Western Blotting

RK13 cells were (co-)transfected with plasmids expressing the indicated gH variants with or without the gL-expression plasmid using Lipofectamine 2000 (Thermo Fisher Scientific), and pEGFP-N1 (Clontech) served as negative control. For virus characterization, RK13 cells were infected with the different mutants and PrV-Ka at a MOI of 3. Transfected and infected cells were harvested one day later, washed with phosphate-buffered saline (PBS), and lyzed in sample buffer (0.13M Tris-HCl, pH 6.8; 4% SDS; 20% glycerol; 0.01% bromophenol blue; 10% 2-mercaptoethanol). After boiling for 3 min, proteins were separated on SDS-10% or 12% polyacrylamide gels and transferred to nitrocellulose. Membranes were blocked with 5% skimmed milk in tris-buffered saline with 0.1% Tween-20 (TBS-T), and probed with monospecific PrV antisera diluted in TBS-T (anti-gH 1:15.000, anti-gL 1:1.000, anti-UL38 1:100.000) [7,9] or an anti-tubulin monoclonal antibody (Sigma-Aldrich, Taufkirchen, Germany) as loading control. Bound antibody was detected after incubation with horseradish peroxidase-conjugated α-rabbit or α-mouse IgG (Invitrogen, Waltham, MA, USA) using the Clarity Western ECL Substrate (BioRad). Signals were recorded with a Versa DOC 4000 MP imager (BioRad, Feldkirchen, Germany).

### 2.7. Semiquantitative Analyses of gH Surface Expression

Semiquantitative analyses were performed as described previously [31]. Briefly, RK13 cells transfected with equal amounts of the different gH expression plasmids were fixed one day post transfection with 3% paraformaldehyde and were either permeabilized with 0.3% Triton X-100 for 10 min or left untreated. Mean fluorescence intensities after incubation with the monospecific anti-gH serum [9] and Alexa Fluor^TM^ 488 goat anti-rabbit IgG were measured by microscopy (Eclipse Ti-S with the software NIS-Elements, version 4.0; Nikon, Tokyo, Japan) using the 10× objective in six fields of view. Cells transfected with the empty vector pcDNA3 served as background control and cells co-transfected with gH Ka and gL served as 100% value. Percentages of gH-specific fluorescence were determined after background subtraction and mean values, as well as standard deviations, were calculated from three independent experiments.

### 2.8. Generation of PrV-gH I662S and PrV-gH I662S/ΔgL

A PrV mutant expressing gH I662S was generated by BAC mutagenesis as described [29], using a gfp-expressing, gG and gH gene-deleted, full-length clone of PrV-Ka (pPrV-ΔgHABF), and the gH rescue plasmid pcDNA-gHKDE, which had been modified by the I662S substitution. The gL deleted version was also generated by BAC mutagenesis according to procedures described [32]. Infectious virus was propagated on RK13 cells. Correct insertion of gH I662S and absence of gL was verified by sequencing after PCR amplification of the corresponding gene regions and by Western blot analyses.

### 2.9. Statistical Analyses

The statistical significance of differences was evaluated using unpaired *t* test with Welch’s correction provided by GraphPad Prism 9 software (GraphPad Software, Inc., San Diego, CA, USA).

## 3. Results

### 3.1. Isolation and Characterization of PrV-ΔgLPassV99

Previous passaging experiments in non-complementing cells using phenotypically complemented PrV-ΔgL resulted in revertants, which carried mutations in the N-terminal gL-binding domain of gH. Since the compensatory mutations were very different in the previous revertants, i.e., formation of a gD-gH hybrid protein in the first passaging experiment [9] and two amino acid changes in the gH N-terminus in the second [8], we wanted to investigate whether there are even more ways to overcome the missing gL function and repeated the passaging experiment. Single virus plaques from the 99th passage of PrV-ΔgLgfp were picked and tested for infectivity. One infectious isolate, designated as PrV-ΔgLPassV99, was further characterized and absence of gL was confirmed.

**Figure 1:**
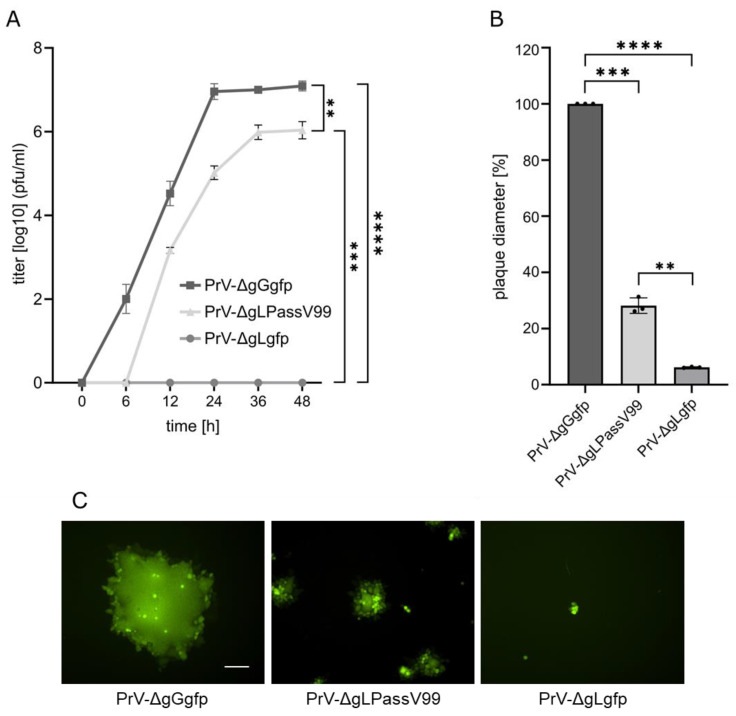
Replication properties of PrV-ΔgLPassV99. (**A**) RK13 cells were infected with PrV-ΔgGgfp, PrV-ΔgLPassV99 and PrV-ΔgLgfp at a MOI of 0.5. Cells and supernatant were harvested at the indicated times after infection and titers were determined on RK13 cells. Mean titers in log10 plaque forming units [pfu]/mL and corresponding standard deviations are given. (**B**) RK13 cells were infected under plaque assay conditions and plaque diameters were measured two days post infection. Shown are mean percent values compared to PrV-ΔgGgfp plaques set as 100% and corresponding standard deviation from three different experiments. Two-tailed Welch’s *t* test; **, *p* < 0.01; ***, *p* < 0.001; ****, *p* < 0.0001. (**C**) Representative images of plaques formed one day post infection. Scale bar: 100 µm.

Multistep growth kinetics (Figure 1A) showed that PrV-ΔgLPassV99 replicated efficiently in RK13 cells and reached titers only 5- to 10-fold lower than the wild type-like PrV-ΔgGgfp, while the parental PrV-ΔgLgfp did not produce infectious progeny on non-complementing cells. Plaque diameters of PrV-ΔgLPassV99 reached only approximately 30% compared to PrV-ΔgGgfp, but were clearly discernible from the small foci of infected cells after infection with PrV-ΔgLgfp (Figure 1B,C). 

Since, in our previously characterized revertants, mutations in one or several of the other entry glycoproteins were found [8,9], genes encoding gB, gD and gH were amplified by PCR from genomic PrV-ΔgLPassV99 DNA and sequenced as described [8]. Mutations resulting in amino acid changes are summarized in Table 2.

No mutation was found in the gD gene, while the gB open reading frame carried two alterations resulting in amino acid substitutions (Table 2). Surprisingly, the N-terminal gL-interaction domain of gH was unchanged, but two mutations in the predicted gH TMD (aa 647-667; [20]) were identified. Alanine at position 651 was changed to threonine (A651T) and isoleucine at position 662 was substituted by serine (I662S) (Table 2).

### 3.2. Mutations in the gH TMD Are Reponsible for gL-Independent Fusion

To test whether the mutations in gB, gH or both are required for gL-independent membrane fusion, expression plasmids encoding the parental PrV-Kaplan (Ka) glycoproteins or the PrV-ΔgLPassV99 variants (V99) were cotransfected into RK13 cells in different combinations with or without addition of pcDNA-gL, and syncytia formation was evaluated 19 h post transfection.

**Figure 2:**
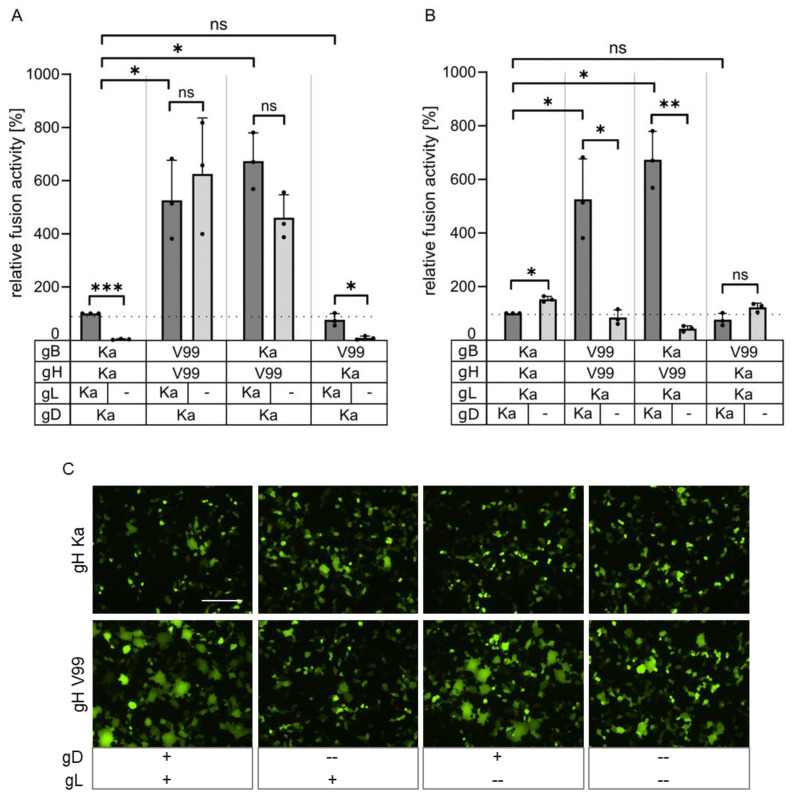
In vitro fusion assays. (**A**) RK13 cells were transfected with expression plasmids encoding gB and gH derived either from PrV-Ka (Ka) or PrV-ΔgLPassV99 (V99), gD Ka and with or without (-) pcDNA-gL. (**B**) Plasmids encoding either wild type gL, gB, gH or gB and gH derived from PrV-ΔgLPassV99 were cotransfected into RK13 cells either with or without (-) pcDNA-gD Ka. Area and number of syncytia was measured 19 h post transfection and calculated corresponding to assays with plasmids expressing Ka gB, gD, gH and gL set as 100%. Shown are mean values of three independent assays with corresponding standard deviations. Two-tailed Welch’s *t* test; ns, not significant; *, *p* < 0.05; **, *p* < 0.01; ***, *p* < 0.001. Values for panels A and B were measured in combined assays but separated for clarity. (**C**) Representative images of syncytia formed after cotransfection of RK13 cells with gB Ka and gH Ka or gH V99 in presence (+) or absence (--) of gD and gL. Scale bar: 200 µm.

Whereas cell–cell fusion could not be observed with the Ka-derived plasmids in the absence of gL, gL was not required to induce fusion with gH V99 and either gB Ka or gB V99 (Figure 2A). The combination of gB V99 and gH Ka resulted only in background levels in the absence of gL, indicating that the mutations in the gH TMD are responsible for the gL-independent phenotype. The mutations in gB V99 (E290K, F709L) had no detectable effect on fusion activity, and values reached close to 100% in combination with gH Ka, gD and gL.

PrV gD is known to have no [8,10,32] or a slightly inhibiting effect on in vitro fusion activity when co-expressed with the native PrV Ka glycoproteins (Figure 2B). In contrast, plasmids expressing gH V99 with gD Ka in combination with gB V99 or gB Ka resulted in approximately 5- to 6-fold higher fusion levels, while fusion activity below 100% was found in the absence of gD (Figure 2B). However, gD-mediated hyperfusion was not observed with gB V99 and gH Ka, indicating that this effect is connected to the presence of gH V99.

### 3.3. The Isoleucine to Serine Substitution in the Transmembrane Domain of gH Is Sufficient to Compensate for Absence of gL

To test whether both mutations in gH V99 are required for the gL-independent membrane fusion plasmids expressing gD Ka, gB Ka and gH Ka, gH V99 or gH carrying only the A651T or gH I662S mutation were cotransfected with or without the gL-expression plasmid and syncytia were analyzed 19 h post transfection.

**Figure 3:**
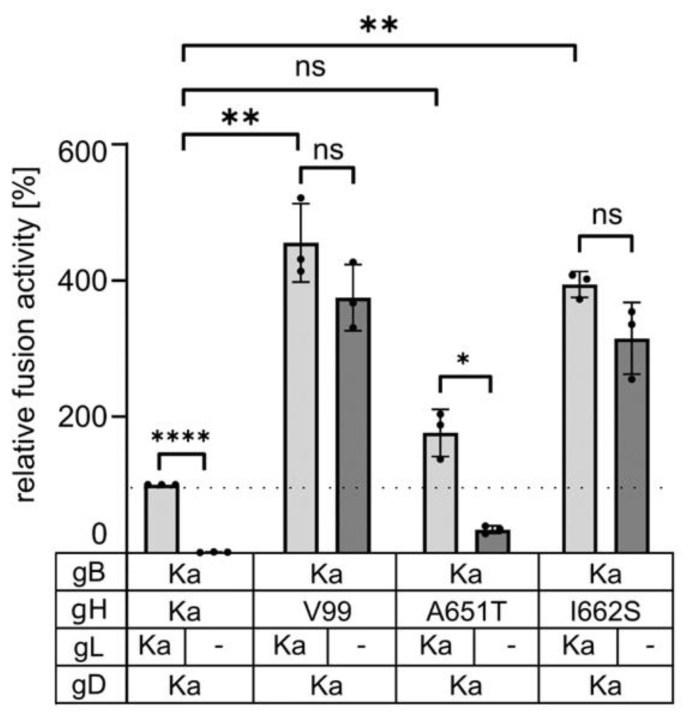
The isoleucine to serine substitution in the gH TMD is sufficient for efficient gL-independent membrane fusion. Plasmids encoding gB Ka, gD Ka, gH Ka, gH V99 or gH carrying only one substitution were cotransfected into RK13 cells either in presence or absence of pcDNA-gL and syncytia were measured 19 h post transfection. Shown are mean results of three independent assays with the corresponding standard deviation with values for assays with gB Ka, gH Ka, gD and gL set as 100%. Two-tailed Welch’s *t* test; ns, not significant; *, *p* < 0.05; **, *p* < 0.01; ****, *p* < 0.0001.

Co-expression of gB Ka with gH I662S induced efficient cell–cell fusion in the absence of gL, while gL-independent fusion with gH A651T reached only 34% of the gL-dependent activity of gH Ka identifying the I662S substitution as the major factor for gL-independent fusion (Figure 3). However, compared to gH Ka, gH A651T resulted in moderately increased fusion levels in presence and absence of gL, indicating that also this substitution in the gH TMD influences fusion regulation.

### 3.4. Serine, Alanine, Cysteine and Threonine at Position 662 in the gH Transmembrane Domain Can Efficiently Compensate for gL Function in Cell–cell Fusion

We next asked whether the gL-independent phenotype is specific for the serine substitution in the gH TMD. Therefore, we changed the isoleucine codon 662 to codons for different other amino acids by site directed mutagenesis. Successful mutagenesis was verified by sequencing.

**Figure 4:**
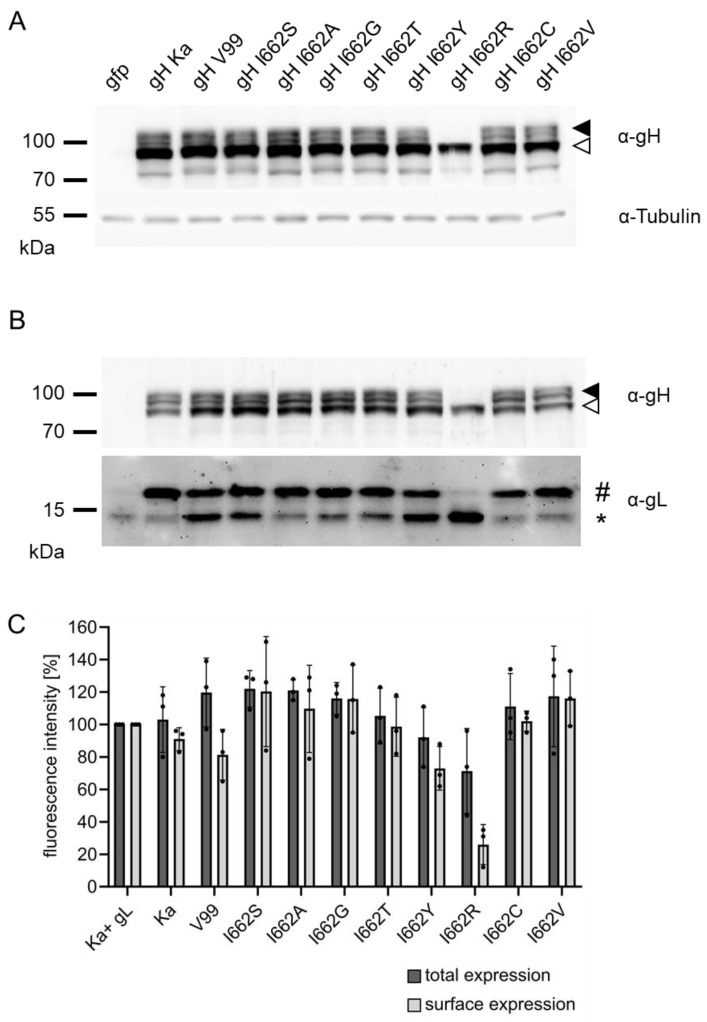
Expression and processing of the different gH variants. (**A**) RK13 cells were transfected with expression plasmids for gH Ka or the different gH mutants and cell lysates were harvested one day post transfection. After Western blotting, the membrane was cut between the 70 and 55 kDa markers and the upper part was probed with a monospecific rabbit α-gH serum and the lower part with an anti-tubulin monoclonal antibody as loading control. (**B**) RK13 cells were cotransfected with expression plasmids for gL and the different gH variants. Parallel blots were probed with anti-gH and anti-gL sera. Immature and mature forms of gH are indicated by open and filled arrow heads, and of gL by an asterisk (*) and a diamond (#), respectively. Cells transfected with pEGFP-N1 were used as negative control. Molecular masses of marker proteins in kDa are given. (**C**) Relative total or cell surface fluorescence intensities of cells transfected with the indicated expression constructs are given. Cells cotransfected with pcDNA-gH Ka and pcDNA-gL were set as 100%, and cells transfected with the empty vector were used as background control. Mean values of three independent assays and standard deviations are shown.

Correct expression of the gH variants was analyzed by transfection of RK13 with the corresponding expression plasmids and subsequent Western blotting. As shown in Figure 4A, all gH variants showed expression levels and glycosylation patterns comparable to gH Ka, except for gH I662R, which was not detectably processed. Maturation of gH I662R could also not be observed after cotransfection with pcDNA-gL (Figure 4B), indicating that this mutation most likely resulted in misfolding and retention of the protein in the endoplasmic reticulum. In line, the gH I662R mutant could not be detected at the surface of transfected, nonpermeabilized cells (Figure 4C). Therefore, this construct was excluded from further assays. Semiquantitative analyses of the total and surface expression of the different gH versions assayed by indirect immunofluorescence after transfection (Figure 4C), showed a slight but non-significant increase in surface expression of gH mutants with exchange of I662 to S, A, G, C or V.

**Figure 5:**
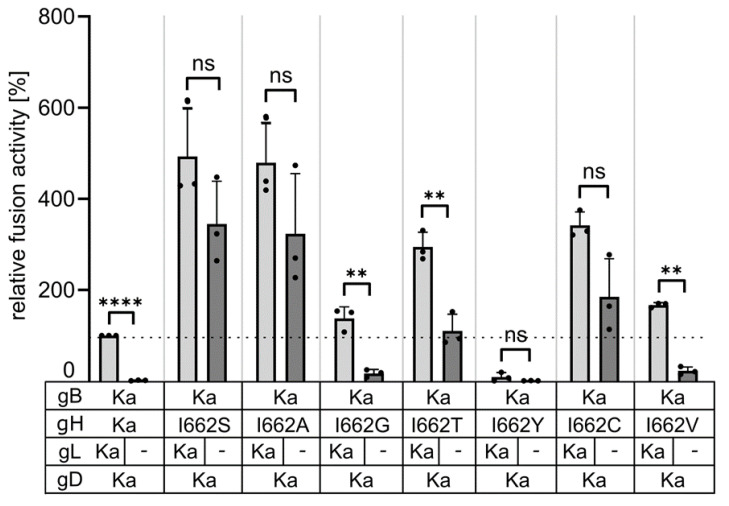
Different amino acids at position 662 in the gH TMD compensate for lack of gL in cell–cell fusion assays. Expression plasmids encoding gD Ka, gB Ka, gH Ka or mutated gH as indicated were cotransfected either in presence (black bars) or absence (grey bars) of pcDNA-gL into RK13 cells. Relative fusion activity was determined 19 h post transfection. Mean values of three independent assays and corresponding standard deviation are given. Two-tailed Welch’s *t* test; ns, not significant; **, *p* < 0.01; ****, *p* < 0.0001.

To test for gL-independent cell–cell fusion, plasmids expressing gB Ka, gD Ka, and gH Ka or the different gH mutants were cotransfected into RK13 cells, together with or without pcDNA-gL. All gH mutants with the exception of gH I662Y showed elevated fusion activity compared to gH Ka in presence of gL, gD and gB Ka. Efficient cell–cell fusion in presence and in the absence of gL was detectable with gH I662S, gH I662A, gH I662T and gH I662C. Glycine (G) or valine (V) at this position conferred only low levels of gL-independent cell–cell fusion, while gH Ka with the native isoleucine at this position was unable to induce syncytia in the absence of gL. gH I662Y induced only very low levels of fusion in the presence of gL, and was nonfunctional without gL (Figure 5).

### 3.5. gH I662S Is Sufficient to Compensate for gL Function during Virus Entry

**Figure 6:**
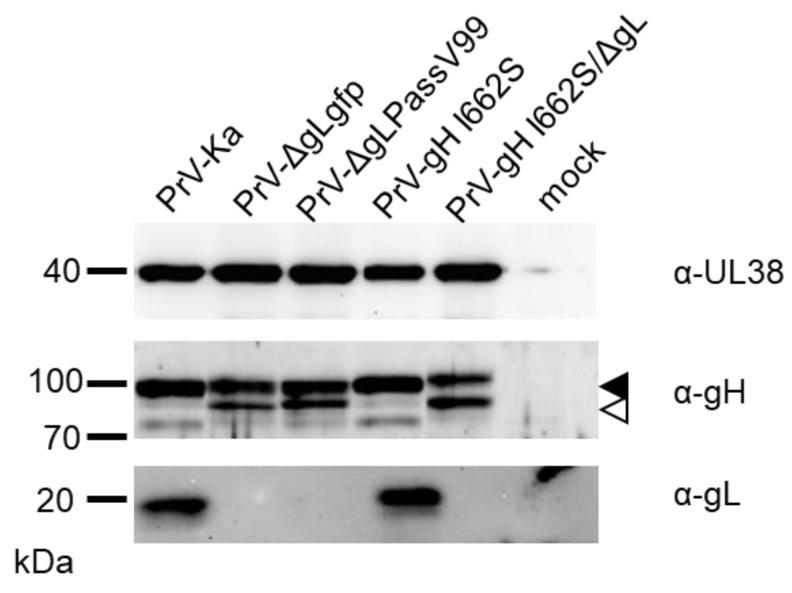
Western blot analysis of RK13 cells infected with PrV-Ka or the indicated mutants and uninfected control cells (mock). Cells were infected at a MOI of 3 and harvested one day post infection. Cell lysates were separated in SDS-10% or 12% polyacrylamide gels and membranes were incubated with monospecific anti-gH and anti-gL sera as indicated. Immature and mature gH forms are indicated by open and filled arrow heads, respectively. An anti-pUL38 (capsid protein) serum was used for infection control. Molecular mass markers (in kDa) are given on the left.

In vitro transfection based fusion assays are a well-established and valuable surrogate model to investigate membrane fusion in the absence of virus infection. However, to study directly whether gH I662S is also able to complement gL function during virus entry, we generated a PrV-Ka mutant expressing gH I662S, as well as a gL-deleted version thereof. DNA sequence analyses and Western blot analyses (Figure 6) verified the absence of gL in the corresponding virus mutants.

The approximately 18–20 kDa gL was only detectable in lysates of cells infected with PrV-Ka and PrV-gH I662S. Absence of gL results in a less efficient processing of gH [9], which was confirmed by stronger signals for the immature relative to the mature gH in PrV-ΔgLgfp, PrV-ΔgLPassV99 and PrV-gH I662S/ΔgL (Figure 6).

**Figure 7:**
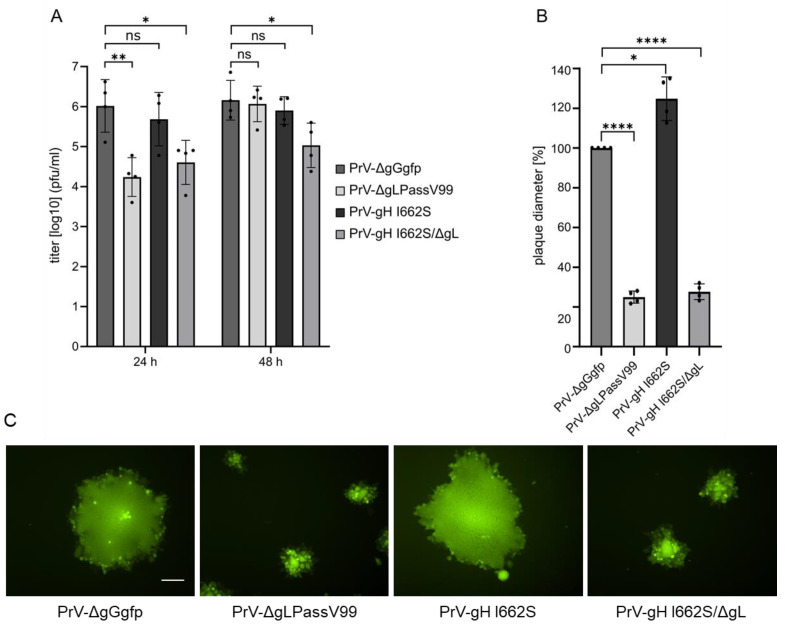
Growth properties of PrV-gH I662S and PrV-gH I662S/ΔgL. (**A**) RK13 cells were infected with the indicated mutants at a MOI of 0.5. Cells and supernatant were harvested at 24 h and 48 h post infection, and titers were determined on RK13 cells. Mean titers and corresponding standard deviations of three independent experiments are shown. (**B**) RK13 were infected with the indicated virus mutants under plaque assay conditions and plaque diameters were measured two days post infection. Shown are mean percent values (compared to PrV-ΔgGgfp plaques) and corresponding standard deviations from three different experiments. Two-tailed Welch’s *t* test; ns, not significant; *, *p* < 0.05; **, *p* < 0.01; ****, *p* < 0.0001. (**C**) Representative images of plaques formed by the indicated virus mutants one day post infection are shown. Scale bar: 100 µm.

The growth properties of the mutants were analyzed after infection of RK13 cells. As shown (Figure 1), PrV-ΔgLPassV99 replicated to lower viral titers than PrV-ΔgGgfp, in particular at early times (24 h post infection). This delay in replication was not observed with PrV-Ka expressing gH I662S (Figure 7A), and is presumably due to a delayed virus penetration in absence of gL [9]. Titers of PrV-gH I662S/ΔgL were similar to PrV-ΔgLPassV99 at 24 h post infection and only less than 10-fold reduced after 48 h (Figure 7A), indicating that the gH I662S substitution is sufficient to promote gL-independent entry and productive replication.

However, in contrast to efficient formation of viral progeny, cell–cell spread of PrV-gH I662S/ΔgL and PrV-ΔgLPassV99 was reduced to a similar extent, while the gH I662S substitution in the wild-type background slightly increased plaque diameters (Figure 7B). Plaque morphology of PrV-ΔgGgfp and PrV-gH I662S on the one hand, and of PrV-gH I662S/ΔgL and PrV-ΔgLPassV99 on the other hand, was comparable (Figure 7C).

## 4. Discussion

The complex mechanism mediating membrane fusion of herpesviruses for infectious entry and direct cell-to-cell transmission is still not fully understood. Although structural information of the viral entry glycoproteins complements a plethora of functional data obtained from different molecular biological and biochemical assays, the membrane fusion activation of gB triggered by gH/gL after receptor binding still remains enigmatic.

We repeatedly used an indirect approach to uncover the functional role of the core fusion glycoproteins by forcing the virus to adapt through in vitro viral evolution of respective deletion mutants. PrV exhibits a broad host range in vitro and in vivo, and appears more promiscuous in its requirements for entry and spread compared to other herpesviruses. In vitro evolution by serial co-passaging of infected with non-infected cells already resulted in the isolation of mutant viruses that are capable of infectious entry without the receptor binding glycoprotein gD [35], part of the fusion regulatory gB CTD [24,36] or gL [8,9]. Except for the formation of the gD-gH hybrid protein, most of the compensatory mutations comprised only minor changes in the amino acid sequences in the remaining components of the core fusion apparatus pointing to an intrinsic flexibility within the fusion machinery, at least in PrV.

The previously characterized gL-independent infectious revertants carried mutations in the N-terminal domain of gH [8,9], which comprises the gL-interaction domain [5], indicating that gL may maintain gH in an inactive state prior to release by receptor-activated gD. This gL function could obviously be compensated by two amino acid substitutions [8] or a deletion in the gH N-terminus (codons 32–98 [32]). In contrast, in PrV-ΔgLPassV99, the N-terminal part of gH was unaffected but the predicted TMD carried two amino acid substitutions, A651T and I662S. The isoleucine to serine substitution at position 662 was sufficient to induce efficient membrane fusion in the absence of gL (Figure 3), while the amino acid changes identified in gB V99 (E290K, F709L) had no detectable effect on in vitro membrane fusion activity (Figure 2) or for entry, replication and direct cell-to-cell transmission in the viral background (data not shown).

In contrast to in vitro fusion assays with the wild type (Ka) PrV glycoproteins where presence of gD had no fusion-enhancing effect [10,32], significant gL-independent hyperfusion was observed with gH V99 in the presence of gD (Figure 2). Protein complexes between gD and gH/gL, gD and gB, as well as between gB and gH/gL in presence of gD, have been identified in HSV-1 by bimolecular complementation assays [16,37,38,39], supporting the prevalent cascade-like interaction and activation model for gB-mediated fusion [14]. In a recently proposed model, however, it is suggested that all components of the fusion machinery already interact before fusion occurs, with gH/gL being positioned between gD and gB thereby separating the interaction from the activation function [16]. Up to now, direct interactions between the core fusion glycoproteins have not been experimentally verified for PrV. However, presence of gD might further stabilize the supposed interaction between the gH V99 molecules or between gH V99 and gB, allowing for enhanced cell–cell fusion activity. Further experimentation is required to elucidate the molecular details of the gD-driven hyperfusion in the presence of gH V99.

**Figure 8:**
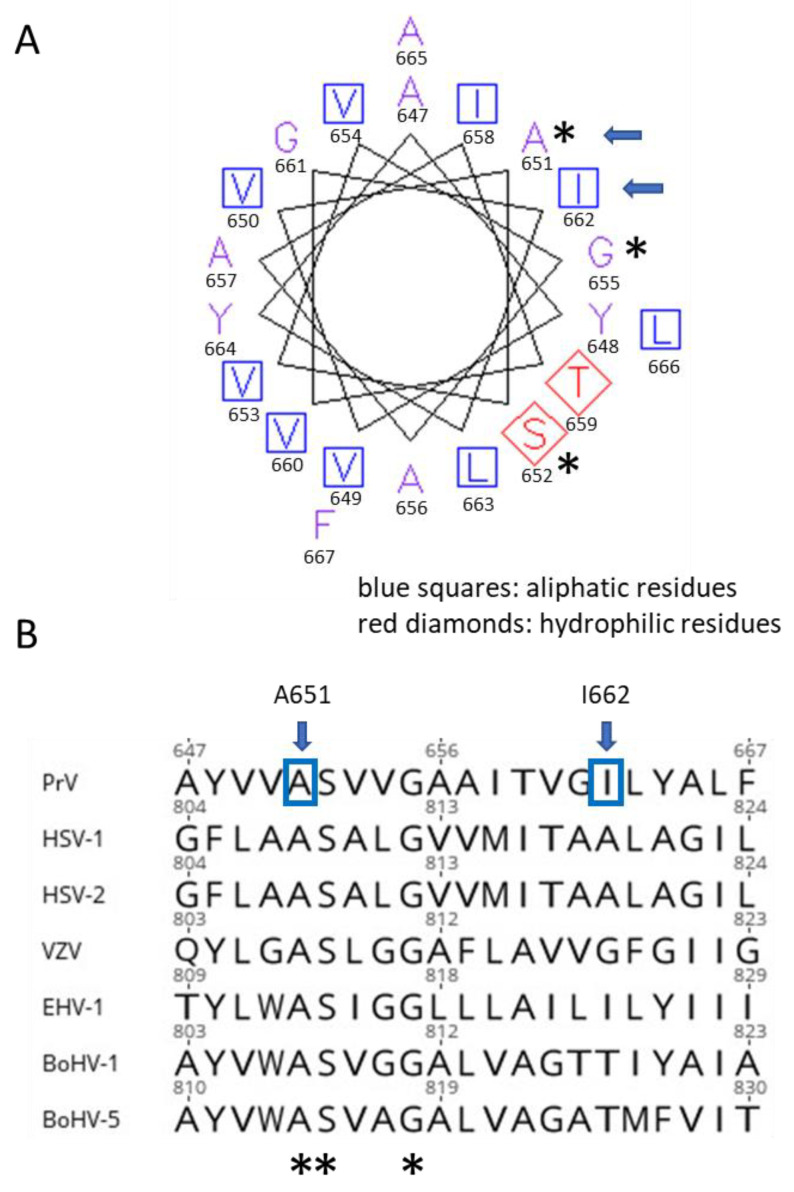
(**A**) Helical wheel plot (EMBOSS: pepwheel (bioinformatics.nl)) of the PrV gH TMD (aa 647–667) and (**B**) alignment of alphaherpesvirus gH transmembrane domains corresponding to Harman et al. [21]. Conserved residues and amino acid substitutions in the PrV gH V99 TMD localize on the same face of the predicted alpha helix. Residues substituted in the gH V99 TMD are marked by blue arrows, and the conserved amino acids are highlighted by asterisks (*). HSV-1: YP_009137096, HSV-2: YP_009137173 VZV: varicella zoster virus (*Varicellovirus humanalpha3*) CAA27920; EHV-1: equine herpesvirus 1 (*Varicellovirus equinealpha1*) AAS45924, BoHV-1: bovine herpesvirus 1 (*Varicellovirus bovinealpha1*) P27599; BoHV-5: bovine herpesvirus 5 (*Varicellovirus bovinealpha5*) AAD40580.

The helical wheel plot of the gH TMD (Figure 8A) shows that both substituted amino acids in the TMD of gH V99 are located next to each other facing the same side as the highly conserved residues [21] (Figure 8B). This indicates that this side of the gH TMD might constitute the functionally important face of the alpha helix. The substitution at position 651 even affects the conserved alanine residue. Based on interaction studies of membrane-spanning proteins, it is likely that this side is important for TMD-TMD interactions [40]. Thus, TMD-TMD interactions between the fusion glycoproteins might play a role for fusion regulation. Unfortunately, TMD interactions in the membrane are still difficult to assess experimentally.

Still unknown is whether the gH/gL complex acts as a single heterodimer or whether heterooligomers are the functional entity. gH-gH self-interaction has been observed in bimolecular complementation assays for HSV-1, and it was speculated that indeed gH/gL oligomers partner with the gB trimers [39]. In addition, quantitative studies showed that approximately 10-times more gH compared to gB is present on the HSV-1 virion surface [41] which appears sufficient to enclose and shield the gB trimers in the membrane.

In PrV gH, the A651T exchange increased cell–cell fusion (~176% compared to gH Ka) in the presence, but also in the absence, of gL (~34% versus 1% with gH Ka; Figure 3), indicating that the conversion of a hydrophobic to a polar residue at least partly contributes to the gL-independent phenotype of gH V99. Conversely, substitution of the corresponding residue in HSV-1 gH (=A808) by tryptophan reduced fusion activity in transient assays by approximately 50% [21], pointing to species-specific differences and highlighting the importance of this residue in the TMD for fusion.

The major effector of the gL-independent cell–cell fusion, however, is the I662S substitution. Fusion activity induced with gH I662S was only slightly lower, as with gH V99 (Figure 3). In the helical wheel plot, I662 is located between the conserved alanine and glycine residues (Figure 8). The glycine residue in HSV-1 gH was found to be particularly sensitive to substitutions and in in vitro fusion assays; this residue could be functionally substituted by amino acids with small side chains (A, S, C, T, V), but not by more bulky residues as isoleucine, leucine or methionine, pointing to a steric hindrance [21]. The residue in HSV-1 gH (A819) corresponding to PrV gH I662 was unfortunately not addressed, and the functional importance of gL was not investigated.

Similar to the conserved glycine residue in HSV-1 gH, isoleucine at position 662 in PrV gH could be functionally substituted by several amino acids with small side chains (A, S, C, T) and efficient cell–cell fusion in the presence, but also in the absence, of gL was observed (Figure 5). Glycine and valine at position 622 still allowed for higher fusion levels compared to gH Ka (Figure 3), while substitution by tyrosine with its large phenol ring impaired cell–cell fusion despite proper protein expression (Figure 4). A clear correlation between fusion activity (Figure 5) and cell surface expression of the gH mutants (Figure 4C) was not evident. gH I662R was nonfunctional (data not shown) since its processing and cell surface expression was severely impaired (Figure 4). Taken together, these results indicate that the native isoleucine, as well as probably other bulky amino acids at position 662 in PrV gH, negatively act on fusion activation. In contrast, smaller residues might allow for a greater flexibility or a tighter packing of the TMDs of the fusion complex proteins within the membrane, since it has been shown that small and weakly polar residues allow TMDs to come into closer contact [42]. The enhanced flexibility and/or tighter TMD packing may induce a gH conformational change by long-range allosteric effects or a change in the overall TMD dynamics eventually resulting in the downstream conformational switch of the gB ectodomain. However, it remains unclear why gH molecules with glycine or valine at position 662, which possess also small side chains mediate only very low levels of gL-independent fusion (Figure 5). It cannot be excluded that the mutations might also have a direct effect on the local lipid environment, which might influence the energy barrier to fusion.

The gH I662S substitution not only compensates for gL function in in vitro fusion assays, but also during virus entry and replication (Figure 7A). A virus recombinant expressing gH I662S reached comparable titers as the wild type-like PrV-ΔgGgfp, and the isogenic mutant lacking gL, PrV-gH I662S/ΔgL, replicated to only approximately 10-fold lower titers, whereas non-passaged PrV-ΔgL was unable to replicate on non-complementing cells (Figure 1). This indicates that the amino acid substitution I662S in the gH TMD is the dominant effector of the revertant phenotype also in the virus background. The mutations in gB V99 (E290K, F709L) seem to also play no major role in the virus background. 

The in vitro fusion assays usually more closely mimic the fusion process during cell-to-cell transmission than during entry [10]. Here, we found a clear difference: whereas V99 gH mediates gL-independent hyperfusion in the presence of gB and gD in vitro (Figure 2), virus mutants deficient for gL but expressing gH V99 (PrV-ΔgLPassV99, PrV-gH I662S/ΔgL) form only small plaques (Figure 7B,C). As evident from immunoblots (Figure 6), processing of gH is less efficient in the absence of gL, and it might be speculated that transport of gH to the sites of cell–cell-spread is impaired, regardless of the TMD mutations in the absence of gL.

In summary, our data uncovered an important role of the gH TMD in fusion regulation, expanding our knowledge about the structural elements involved in herpesvirus fusion. Additional investigations are imperative to fully elucidate the precise regulatory function governed by the gH TMD. However, our findings support different prospective models for the role of the gH TMD: (i) the gH TMD is implicated in intramolecular signaling of gH, enabling allosteric coupling between the gH ectodomain and the gH CTD, thereby facilitating the release of the gB CTD for fusion; (ii) the gH TMD partakes in TMD-TMD interactions among glycoproteins important for membrane fusion; (iii) mutations in the gH TMD might act on the local lipid environment thereby influencing membrane fusion probability.

Timely fusion activation obviously engages all structural parts of the core fusion glycoproteins and is controlled by more than one safety mechanism. Future research focusing on the exact nature of the regulatory function exerted by the gH TMD and the exact stoichiometry of the fusion machinery “in action” will help to understand the complex interplay between the different components in more detail.

## Figures and Tables

**Table 1:** Primers used for site-directed mutagenesis.

Primer Designation	Primer Sequence (5′ -> 3′) ^1^
gH A651T	C GTG GTG ACC TCC GTC GTG G
gH I662A	CG GCC ATC ACC GTG GGG GCC CTG TAC GCC CTA TTC
gH I662G	CG GCC ATC ACC GTG GGG GGC CTG TAC GCC CTA TTC
gH I662R	CG GCC ATC ACC GTG GGG CGC CTG TAC GCC CTA TTC
gH I662S	CG GCC ATC ACC GTG GGG AGC CTG TAC GCC CTA TTC
gH I662T	CG GCC ATC ACC GTG GGG ACC CTG TAC GCC CTA TTC
gH I662Y	CG GCC ATC ACC GTG GGG TAC CTG TAC GCC CTA TTC
gH I662C	CG GCC ATC ACC GTG GGG TGC CTG TAC GCC CTA TTC
gH I662V	CG GCC ATC ACC GTG GGG GTC CTG TAC GCC CTA TTC

^1^ Nucleotides exchanged are underlined; only the forward primers are given.

**Table 2:** Amino acid changes in the entry glycoproteins of PrV-ΔgLPassV99.

gB	gD	gH
E290K	none	A651T
F709L		I662S

Amino acid changes are given in one letter code and the position in the sequence of PrV gB Ka accession number AEM64049.1 [33] or gH Ka accession number JQ809328 [34].

## Data Availability

The raw data are available on request from the corresponding author.
